# Public awareness of ear health and hearing loss in Jeddah, Saudi Arabia

**DOI:** 10.4102/sajcd.v66i1.633

**Published:** 2019-08-26

**Authors:** Khalid A. Alshehri, Waleed M. Alqulayti, Bassam E. Yaghmoor, Hisham Alem

**Affiliations:** 1 College of Medicine, King Abdulaziz University, Jeddah, Saudi Arabia; 2 Department of Otorhinolaryngology, College of Medicine, King Abdulaziz University, Jeddah, Saudi Arabia

**Keywords:** Public awareness, ear health, hearing loss, audiology, Saudi Arabia

## Abstract

**Background:**

Knowledge of ear health and hearing loss is essential for early intervention and treatment, but the state of public knowledge of such in Jeddah, Saudi Arabia, remains unknown.

**Objectives:**

To measure public knowledge of ear health and hearing loss.

**Method:**

This cross-sectional study was conducted during August 2018 in Jeddah, Saudi Arabia. An electronic survey questionnaire was distributed to 2372 residents of various districts in Jeddah. The survey was created in 2013 by adopting previously published World Health Organization material, designed to assess knowledge and perception of ear health. All males and females aged 10 years or older living in Jeddah had the chance to participate in this study. A total of 2372 people participated. Percentages mean ± standard deviation, one-way analysis of variance (ANOVAs) and independent *t*-tests were applied to determine the significant difference. *p*-values of 0.05 or less were considered significant.

**Results:**

The overall mean number of correct responses on the survey’s true/false questionnaire was 10.66 ± 1.92 out of 14. Female participants gave a higher mean number of correct answers than did male participants (10.73 ± 2.03 vs. 10.54 ± 2.03, respectively). Participants with a family history of hearing loss gave a higher mean number of correct answers compared with participants who reported a negative family history (10.89 ± 1.82 vs. 10.53 ± 1.97, respectively).

**Conclusion:**

Overall awareness of ear health and hearing loss management is fair. However, the results indicate a need for more integrated educational materials to be made available both to the general population as well as to hearing loss individuals and their families.

## Introduction

Hearing loss limits one’s ability to distinguish between sounds correctly, making it challenging to understand basic conversations. Furthermore, hearing loss in early life can significantly impair both development and educational achievement, thus negatively impacting both emotional and social quality of life (Agrawal, Platz, & Niparko, 2009; World Health Organization [WHO], 2012).

Many factors throughout all stages of life can cause hearing loss. In particular, chronic ear infection, which can be prevented and managed with medical and surgical interventions, remains one of the leading causes of hearing loss (WHO, 2012). Other causes include exposure to loud noises, use of ototoxic medication and diseases such as meningitis, measles, mumps and rubella (WHO, 2012).

The WHO estimates that there are 466 million individuals worldwide with moderate to profound bilateral hearing loss, two-thirds of whom live in developing countries and suffer from preventable forms of hearing loss (WHO, 2012, 2017). The highest prevalence of disabling hearing loss in both children and adults can be found in South Asia, Asia-Pacific and sub-Saharan Africa (WHO, 2012). This geographical distribution represents a widespread healthcare problem in that many individuals with impaired hearing are not able to access the facilities they require. These difficulties arise from a variety of issues, including limited resources, a paucity of specialised healthcare professionals, poor professional and public awareness, and natural and geographical barriers (Swanepoel et al., 2010). These problems are not only limited to isolated rural regions or developing countries but also exist in urban communities (Swanepoel et al., 2010).

As public knowledge about hearing loss and ear health is essential for prevention and early treatment, several studies have been conducted to estimate public awareness of ear diseases and their management. For example, a study conducted in Italy found that female participants have significantly more knowledge about non-verbal individuals with severe hearing loss and about using a cotton bud for cleaning than male (Di Berardino et al., 2013). Another study conducted in Greece concluded that suitable educational programmes could enhance the ability of nurses to communicate with deaf people (Velonaki et al., 2015). In a striking example of the importance of education efforts, a study published in India found that parents who were unfamiliar with the topic of hearing loss were less likely to know that late response to speech and late speech development could be associated with hearing loss (Merugumala, Pothula, & Cooper, 2017). Finally, a South African study concluded that lack of public awareness regarding audiologists and their profession could affect both hearing health and hearing-related medical care (Joubert, Sebothoma, & Kgare, 2017).

Many patients who are in danger of hearing loss can be effectively treated if intervention takes place early enough. To the best of our knowledge, there are limited data on the general public’s awareness of ear health and hearing loss in Saudi Arabia. This study aimed to measure such awareness among the population of Jeddah, Saudi Arabia, in 2018.

## Methods

### Study design and objective

A quantitative cross-sectional study aimed to measure such awareness among the population of Jeddah, Saudi Arabia, in 2018.

### Study setting and participants

This study was conducted in the urban city of Jeddah, Saudi Arabia, during August 2018. Jeddah is one of the largest cities in Saudi Arabia, located in the western region, on the coast of the Red Sea. An electronic questionnaire was distributed by trainee data collectors to residents of different districts of the city. Children under the age of 10 years have been excluded from the study because of their inability to read and write, yielding a realistic sample of the population of Jeddah. Participants were recruited by stratified sampling for each administrative area, which were considered as independent strata. The distribution of the sample size between strata was matched to the percentage of each administrate area to the whole population. Instruction on the purpose of the study and written informed consent were required before filling out the questionnaire. The participants were able to access the survey link through their phones.

### Questionnaire

The self-administered electronic survey consisted of two parts. The first part comprised questions about demographic and clinical characteristics of the participants, including gender, age, nationality, previous diagnosis of hearing loss, family history of hearing loss, self-reported exposure to noise (listening to loud music, working in factories with loud noise or using headphones loudly) and smoking. The second part of the survey was designed to assess the knowledge and perception of ear health and hearing management. This questionnaire was created in 2013 by adopting previously published WHO material (Di Berardino et al., 2013) and consists of 14 items divided into four domains (knowledge of infant hearing loss, management of ears including cleaning and treating, effects of overexposure to noise and underestimated ear symptoms leading to diagnostic delay) (Table 1). The participants were instructed to evaluate each sentence from the questionnaire as either true or false. The original English questionnaire was translated into Arabic by two independent native Arabic doctors with high proficiency in English. The Arabic version was then translated back into English by two independent native English doctors with high proficiency in Arabic, and the authors compared the back-translated version with the original English version and reconciled all differences.

**TABLE 1:** Questionnaire to evaluate public awareness of ear and hearing management.

Domain	Item	Correct response
Infant hearing loss	1. It is possible to diagnose deafness in infants shortly after birth	True
	2. A deaf–mute cannot speak because of defects in the vocal tract	False
	3. Hearing loss may cause attention deficits thus reducing school performance	True
Cleaning and treating	4. Cotton buds are necessary for ear cleaning and are the safest means	False
	5. Ear drops are sufficient to treat earache	False
	6. Otomycosis (itchy ears) can be contracted at the swimming pool	True
	7. Drug abuse does not provoke auditory hallucinations or modifications of hearing quality	False
	8. Hearing aids need to fit accurately to provide the maximum benefit	True
Physical agents and overexposure	9. Kisses or slaps on the ears do not cause hearing problems	False
	10. Listening to music for more than 3 h a day using earphones may cause permanent hearing loss	True
	11. There are no tables recommending a reduction in the duration of exposure to high-intensity noises	False
Diagnostic delay	12. Irritating perception of sound (e.g. hearing metallic voices) and/or a reduction in hearing clarity (such as a sensation of having cotton wool in the ears) require medical advice	True
	13. Sudden hearing loss is an emergency and requires an immediate audiological assessment	True
	14. Age-related hearing loss may affect behaviour	True

*Source*: Di Berardino, F., Forti, S., Iacona, E., Orlandi, G. P., Ambrosetti, U., & Cesarani, A. (2013). Public awareness of ear and hearing management as measured using a specific questionnaire. *European Archives of Oto-Rhino-Laryngology, 270*(2), 449–453. https://doi.org/10.1007/s00405-012-1961-3

### Data collection and analysis

Data were collected in an electronic Google form and then transformed to the Statistical Package for Social Sciences (SPSS) software version 22 (IBM Corp., Armonk, NY), which was used to code and analyse all the data. All identifying information from the participants was removed to ensure privacy and confidentiality, and only the authors had access to the data. Categorical variables are presented as percentages, while continuous variables are presented as mean ± standard deviation. One-way analysis of variance (ANOVAs) and independent *t*-tests were used to assess the significant difference between the results of the questionnaire and the demographic and clinical characteristics. For all statistical tests, *p*-values of 0.05 or less were considered significant.

### Ethical consideration

This study was approved by the Institutional Review Board (IRB) of King Abdulaziz University Hospital. All identifying information from the participants was removed to ensure privacy and confidentiality, and only the authors had access to the data. Ethical Clearance number is 510-18.

## Results

Out of 5648, 2372 people agreed to participate in our study, reflecting a response rate of 42%. Of these, 64.7% were women and 35.3% were men. The mean age was 31.31 ± 11.85 years. A total of 45.1% of participants fell within the age range of 19–29 years. Most of the members of our sample were Saudi (90.1%). The percentage of participants who reported that they had been previously diagnosed with hearing loss and had a positive family history of hearing loss was 5.5% and 37.5%, respectively. Additionally, 35.6% of the respondents stated that they had been exposed to potentially harmful noise. Demographic characteristics of the sample are presented in Table 2.

**TABLE 2:** Demographic characteristics of the sample (*n* = 2372).

Variables	*n*	%	Mean ± s.d.
Age	-	-	31.31 ± 11.85
**Age groups**			
≤18 years	181	7.6	-
19–29 years	1069	45.1	-
30–39 years	486	20.5	-
>40 years	636	26.8	-
**Gender**			
Male	837	35.3	-
Female	1535	64.7	-
**Nationality**			**-**
Saudi	2137	90.1	-
Non-Saudi	235	9.9	-
**Diagnosed with hearing loss**			
Yes	131	5.5	-
No	2241	94.5	-
**Family history of hearing loss**			
Yes	889	37.5	-
No	1483	62.5	-
**Exposure to noise**			
Yes	845	35.6	-
No	1527	64.4	-
**Smoking**			
Yes	332	14.0	-
No	2040	86.0	-

s.d., standard deviation.

There were 14 questions on the true/false questionnaire, and the mean number of correct responses was 10.66 ± 1.92. The eighth question ‘hearing aids need to fit accurately to provide the maximum benefit’ had the highest rate of correct answers (93%), while the 10th question: ‘Listening to music for more than 3 h a day using earphones may cause permanent hearing loss’ had the lowest rate of correct answers (53.4%). The prevalence of correct answers for all questions is shown in Table 3.

**TABLE 3:** The percentage of correct responses to the true or false questionnaire among the sample (*n* = 2372).

Variables	Responses		
	*n*	%	Mean ± s.d.
Overall mean of correct responses			10.66 ± 1.92
**Questions:**			
Infant hearing loss		68.26	-
1. It is possible to diagnose deafness in infants shortly after birth	1684	71.00	-
2. A deaf–mute cannot speak because of defects in the vocal tract	1305	55.00	-
3. Hearing loss may cause attention deficits, thus reducing school performance	1862	78.50	-
**Cleaning and treating**	-	80.06	-
4. Cotton buds are necessary for ear cleaning and are the safest means	1801	75.90	-
5. Ear drops are sufficient to treat earache	1912	80.60	-
6. Otomycosis (itchy ears) can be contracted at the swimming pool	1597	67.30	-
7. Drug abuse does not provoke auditory hallucinations or modifications of hearing quality	1981	83.50	-
8. Hearing aids need to fit accurately to provide the maximum benefit	2206	93.00	-
**Physical agents and overexposure**	-	68.36	-
9. Kisses or slaps on the ears do not cause hearing problems	2034	85.80	-
10. Listening to music for more than 3 h a day using earphones may cause permanent hearing loss	1266	53.40	-
11. There are no tables recommending a reduction in the duration of exposure to high-intensity noises	1563	65.90	-
**Diagnostic delay**	-	85.50	-
12. Irritating perception of sound (e.g. hearing metallic voices) and/or a reduction in hearing clarity (such as a sensation of having cotton wool in the ears) require medical advice	2110	89.00	-
13. Sudden hearing loss is an emergency and requires an immediate audiological assessment	2183	92.00	-
14. Age-related hearing loss may affect behaviour	1792	75.50	-

Note: Values are presented as mean ± s.d. or *n* (%).

s.d., standard deviation.

We compared the mean number of correct answers between various age groups and found that the respondents younger than 18 years had the lowest mean number of correct answers (9.93 ± 2.13), while those aged 40 or older had the highest mean number of correct responses (10.85 ± 1.76). Female participants had a higher mean number of correct answers compared to male participants (10.73 ± 2.03 vs. 10.54 ± 2.03, respectively). Furthermore, Saudi participants had a significantly higher mean number of correct answers compared to non-Saudi participants (*p* = 0.005). Importantly, participants with a family history of hearing loss had a considerably higher mean number of correct answers compared to those who reported no family history (10.89 ± 1.82 vs. 10.53 ± 1.97, respectively). Regarding exposure to noise, people who considered themselves as being regularly exposed to loud noise had a lower mean number of correct answers compared to those who did not (10.59 ± 2.01 vs. 10.71 ± 1.87). Details of the mean number of correct responses within our sample are presented in Table 4.

**TABLE 4:** The mean of correct responses to the true or false questionnaire among the sample (*n* = 2372).

Variables	Responses	
	mean ± s.d.	p*
**Age groups**	-	<0.0001
<18 years	9.93 ± 2.13	-
19–29 years	10.71 ± 1.97	-
30–39 years	10.59 ± 1.87	-
>40 years	10.85 ± 1.76	-
**Gender**	-	0.022
Male	10.54 ± 2.03	-
Female	10.73 ± 2.03	-
**Nationality**	-	0.005
Saudi	10.70 ± 1.90	-
Non-Saudi	10.31 ± 2.08	-
**Diagnosed with hearing loss**	-	0.123
Yes	10.92 ± 1.87	-
No	10.65 ± 1.92	-
**Family history of hearing loss**	-	<0.0001
Yes	10.89 ± 1.82	-
No	10.53 ± 1.97	-
**Exposure to noise**	-	0.145
Yes	10.59 ± 2.01	-
No	10.71 ± 1.87	-
**Smoking**	-	0.12
Yes	10.42 ± 2.03	-
No	10.70 ± 1.90	-

s.d., standard deviation.

* , *p*-values of the age were derived via one-way ANOVA, and *p*-values of the other factors were derived via independent *t*-test.

Multivariate regression analysis revealed that age, nationality, family history of hearing loss and smoking were significantly associated with the true/false questionnaire score. Values are presented in Table 5.

**TABLE 5:** Results of the true or false questionnaire score, based on multivariate analyses.

Variables	*R*	Confidence interval	*p*
Age	0.010	0.004 to 0.017	0.002
Nationality	−0.345	−0.602 to −0.087	0.009
Family history of hearing loss	−0.335	−0.494 to −0.175	<0.0001
Smoking	0.269	0.047 to 0.492	0.017

## Discussion

In this study, we administered a questionnaire on general knowledge of ear health and hearing management and received answers from 2372 residents of Jeddah, Saudi Arabia. Our results show that most answers to each question were correct. The highest correct-answer rate (93%) was for a question about the need for hearing aids to fit correctly to provide maximum benefit, while the lowest correct-answer rate (53.4%) was for a question about whether listening to music for 3 h a day leads to permanent hearing loss, followed by a question about whether vocal cord injury causes deaf–mute persons to be unable to speak (55%). Regarding the questionnaire’s four domains, the percentages of correct answers for each domain varied between 68.26% and 85.5%.

In the current study, we found a strong association between age and level of awareness: participants who were aged 40 years or older were the most likely to answer the questions correctly, while those aged 18 years or younger were the least likely to answer questions correctly. To the best of our knowledge, this observation has not been reported previously. It could be theorised that this association is because of older participants having had more exposure to and interactions with deaf individuals.

There are only a few studies that have assessed awareness of hearing loss in an integrated manner that includes causes, effects and preventive and management measures (Di Berardino et al., 2013; Lass, Woodford, Lundeen, Lundeen, & Everly-Myers, 1986). Interestingly, the question about the need for hearing aids to fit correctly had the lowest correct-answer rate in the previous study (35.8%) but the highest correct-answer rate in our study. However, an older study (Lass et al., 1986) that included 71 high-schoolers reported a similarly high percentage of correct answers to this question (82.6%). This might be explained by relatively older age in the former study (Di Berardino et al., 2013) (mean age, 51.83 ± 16.57 years vs. 31.31 ± 11.85 years in our study and high-schoolers in the other study [Lass et al., 1986]) as hearing aids have been widely developed and used more lately in the 20th century (Mills, 2011).

Similarly, according to the results in the work of Di Berardino et al. (2013) women were more likely to answer correctly to questions related to specific items regarding infant hearing loss, hygiene and treatment; the cause of deaf-mutism; cotton bud safety and the importance of properly fitted hearing aids. This difference was attributed to females being more likely responsible for the care of the health of other family members (Di Berardino et al., 2013).

The relationship between family history and awareness was also significant in their results; however, the relationship was reversed (people with affected relatives were less likely to know that ear drops were insufficient to treat earache). Moreover, Di Berardino et al. reported that exposure to noise was not significantly correlated with a lack of awareness. In contrast, we observed that participants who reported being exposed to noise were more likely to correctly answer questions related to physical agents and overexposure (the effects of kisses and slaps and the recommended duration of noise exposure). The findings in our study can be explained by that having a family history and exposure to noise provides a better background of knowledge and experience, while the lack of knowledge in this group in their study (Di Berardino et al., 2013) may be explained by the advanced age, reducing the awareness of the condition of younger affected relatives and exposure to noise. Nevertheless, these factors need to be further assessed considering the age, which is an important factor in hearing loss and the awareness of it.

Although our study shows a fair overall awareness of factors related to hearing loss and ear health, with an average score of 10.66 out of 14, the lack of knowledge in 20% – 50% of the population on some points could possibly have a negative effect on the care and well-being of deaf people, as well as on hearing loss prevention efforts in the general population. For example, a lack of knowledge about the association between deaf-mutism and early hearing loss could delay the diagnosis and treatment. Indeed, a qualitative study on healthcare staff and parents of affected children in India reported that parents were less likely to recognise delayed speech and delayed response to speech in their children if they did not possess specific knowledge regarding the matter, even if they were highly educated in other fields (Merugumala et al., 2017).

Moreover, a lack of knowledge about the association between hearing loss and performance in school may prevent hearing-impaired children from gaining access to proper care and education. Similarly, a lack of awareness about the association between hearing loss and behaviour in elderly people can precipitate social problems for both affected individuals and their families, hinder the role of elderly people in society and prevent the diagnosis and treatment of hearing loss in this population. Many studies have shown associations between elderly deafness, especially if untreated, and diminished physical ability and activity (Chen, Genther, Betz, & Lin, 2014; Solheim, Kværner, & Falkenberg, 2011), life satisfaction (Solheim et al., 2011), quality of life( Mulrow et al., 1990; Kelly, & Atcherson, 2011) and mortality (Karpa et al., 2010). Lack of public knowledge about the effects of extended listening to music via earphones, the harms of cotton buds, the risk of fungal infections in swimming pools and the recommended duration of noise exposure all lead to poor attitudes regarding prevention of hearing loss. The heterogeneity in awareness found in the present study indicates the need for integrated and comprehensive educational materials to be presented to the public. Indeed, Chung, Des Roches, Meunier and Eavey (2005) reported that individuals in their sample showed a willingness to follow proper precautions after receiving recommendations from a doctor and information about the risks of hearing loss. Education on the possible causal role of genetics is also an important goal to help parents decide whether to receive genetic testing, which could contribute to early detection of hearing problems (Palmer, Lueddeke, & Zhou, 2009). Therefore, broad national screening programme to preschool children besides awareness is recommended to detect this problem early.

Our study had several limitations. Firstly, we did not account for the education level of the respondents, although there is some evidence that education affects awareness level (Merugumala et al., 2017).^7^ Studying the influence of education level would also have implications for estimating awareness in small towns and rural areas. Furthermore, the age distribution of the sample was skewed towards younger participants by the method, requiring an electronic form, and because literacy levels are low among the elderly in Jeddah. This could affect the accuracy of our estimations of awareness. Moreover, this study included residents of Jeddah. A future nation-wide survey would better assess awareness of ear health and hearing loss management in the entire Saudi population, including those living in the rural areas. Finally, we did not include healthcare staff or a representative number of deaf individuals. Studies that include healthcare staff and a more representative number of deaf individuals and their families need to be carried out to accurately assess the effect of awareness on the incidence of hearing loss, as well as to assess the psychosocial well-being, access to healthcare and quality of education and work in the deaf population of Saudi Arabia.

## Conclusion

The overall awareness of ear health and hearing loss management in Jeddah is adequate, although our results indicate the need for more integrated educational materials for both the general population and hearing-impaired individuals and their families. Furthermore, the development of a broad national screening programme can significantly contribute to early detection and management of hearing loss.
